# Serum Resolvin E1 Levels and Its Relationship with Disease Activity in Ulcerative Colitis

**DOI:** 10.1155/2019/6258327

**Published:** 2019-02-14

**Authors:** Süleyman Günay, Ferda Taşova, Huriye Erbak Yılmaz, Zehra Betül Paköz, Cem Çekiç

**Affiliations:** ^1^Department of Gastroenterology, Katip Çelebi University, Atatürk Education and Research Hospital, İzmir, Turkey; ^2^Department of Internal Medicine, Katip Çelebi University, Atatürk Education and Research Hospital, İzmir, Turkey; ^3^Department of Biochemistry, Katip Çelebi University, Atatürk Education and Research Hospital, İzmir, Turkey; ^4^Department of Gastroenterology, Tepecik Education and Research Hospital, İzmir, Turkey

## Abstract

**Background:**

Resolvins originate from *ω*-3 PUFA (polyunsaturated fatty acid) precursors and play a role in the resolution of inflammation. The aim of this study was to determine the serum Resolvin E1 levels in patients with ulcerative colitis (UC) and to evaluate the relationship between the serum Resolvin E1 levels and ulcerative colitis disease activity.

**Methods:**

In this observational study, serum samples were collected from 51 patients with UC and 30 healthy controls for the determination of Resolvin E1 levels. Firstly, we compared the serum Resolvin E1 levels between the UC patients and the control group. Subsequently, Resolvin E1 levels were analyzed in patients with active UC and UC in remission. Finally, the correlation between Resolvin E1 and C-reactive protein (CRP) and partial Mayo score (p-MS) was analyzed to determine the efficacy of Resolvin E1 in predicting disease activity.

**Results:**

Serum Resolvin E1 level was determined in the UC group (3126 ± 1413 ng/ml) and in the control group (2758 ± 1065 ng/ml) (*p* = 0.187). Serum Resolvin E1 levels were determined in patients with active UC (3114 ± 1166 ng/ml) and patients in remission (3132 ± 1520 ng/ml) (*p* = 0.749). In the UC group, a low-grade positive significant association was found between Resolvin E1 and CRP (*r* = 0.303, *p* = 0.031). There was no significant association between Resolvin E1 and partial Mayo score (*r* = −0.207, *p* = 0.146).

**Conclusions:**

There was no sufficient evidence that Resolvin E1 was an appropriate inflammatory marker to determine disease activity in UC.

## 1. Introduction

Ulcerative colitis (UC) is a chronic idiopathic inflammatory disease. Although the exact cause is not known, the complex interaction of genetic, environmental, and immunological factors is responsible for the pathogenesis of the disease [1]. Although markers such as fecal calprotectin and C-reactive protein (CRP) have been widely used for the determination of disease activity in UC, predicting mucosal healing and disease progression are still being a problem [2]. In studies on lipid mediators in acute inflammatory response, omega-3 PUFA-derived mediators (E-series resolvins such as Resolvin E1, D-series resolvins, and protectins) are secreted from inflammatory exudate, and these chemical mediators have anti-inflammatory effects by resorption of inflammation [3–5]. Resolvin E1 targets neutrophils, dendritic cells, macrophages, platelets, and adhesion molecules. In animal models, Resolvin E1 has been shown to have a strong protective effect against leukocyte-mediated tissue damage and excessive proinflammatory gene expression in the pathogenesis of inflammatory diseases. In recent studies, Resolvin E1 was shown to play a role in the etiopathogenesis of inappropriate inflammatory responses including rheumatoid arthritis, multiple sclerosis, bronchial asthma, retinopathies, periodontal diseases, and inflammatory bowel disease [6–8]. In addition, Resolvin E1 has been shown to have anti-inflammatory activity in inflammatory bowel diseases induced by 2,4,6-trinitrobenzenesulfonic acid (TNBS) [9].

The aim of this study was to determine serum Resolvin E1 levels in patients with UC and to evaluate the association between serum Resolvin E1 levels and UC disease activity.

## 2. Patients and Methods

### 2.1. Patient Selection

The study included 51 UC patients and 30 healthy controls over the age of 18 years, followed-up in the IBD unit of the İzmir Katip Çelebi University Gastroenterology Department.

### 2.2. Study Design

The demographic data of the patients with UC and healthy controls were recorded. Disease localizations, behavior types, and treatment modalities were recorded. In determining the disease activity of UC patients, partial Mayo scoring was used (p-MS ≤ 1: remission, p-MS ≥ 2: active disease) [10]. Serum Resolvin E1 levels were compared between the UC patients and the control group. Resolvin E1 levels were analyzed in patients with active UC and UC in remission. Finally, the correlation between Resolvin E1, C-reactive protein (CRP), and partial Mayo score (p-MS) was analyzed to determine the efficacy of Resolvin E1 in the prediction of disease activity.

### 2.3. Exclusion Criteria

Patients with active infection and cardiac, hepatic, or renal insufficiency, patients with colectomy due to UC, pregnant women and women during lactation, and patients with concomitant chronic inflammatory disease were not included in the study.

### 2.4. Assessment of Serum Resolvin E1 Levels

A total of 8-10 ml venous blood was collected from UC and control patients. Serum was stored in the deep freezer at -20°C in clean and dry Eppendorf tubes. Hemolyzed and lipemic samples were not included in the study. The patient serum and standard solution were pipetted into the wells coated with Resolvin E1 antibody. The Resolvin E1-HRP conjugate was added to each well and incubated for one hour at 37°C, followed by washes with 350 microliters. After addition of the substrate for the HRP enzyme, the reaction was terminated using H_2_SO_4_ after dark incubation. The absorbance at 450 nm was read in ELISA plate reader, and the concentration was calculated according to the standard absorbance curve. The Human Resolvin E1 ELISA kit (MyBioSource, San Diego, CA, USA, catalog no: MBS744335, lot no: 20150401) was used in BioTek (ELx800, USA) semiautomatic ELISA device.

### 2.5. Statistical Analysis

All programs were performed using SPSS 17.0 package program. The frequency and percentages of categorical variables, mean, and standard deviation or median and minimum-maximum values of continuous variables were calculated as descriptive statistics. The relationship between categorical variables was tested by chi-square or Fisher's precision test, and the relationship between continuous variables was tested by Spearman correlation analysis. The Mann–Whitney *U* test was used to compare the two independent sample means, and the Kruskal-Wallis test was used to compare the means of more than two independent samples. ROC analysis was performed for all inflammatory markers. The confidence level of the study was 95% (*p* < 0.05 was considered statistically significant).

### 2.6. Ethical Considerations

The Ethics Committee of Katip Çelebi University, Turkey, approved this study. After the voluntary consent form was obtained from all patients, the study was initiated.

## 3. Results

Fifty-one patients with UC and 31 healthy controls were evaluated. The mean age of the patients with UC was 43 ± 16.1, and the mean age of the control group was 32.4 ± 9 (*p* = 0.003). Thirty-two (62.7%) of the patients with UC and 11 (36.7%) of the control group were male. There was a statistically significant difference between the UC and control groups in terms of age and gender (*p* = 0.003 and *p* = 0.023, respectively). Demographic and clinical features of UC patients are presented in Table 1.

**Figure 1:**
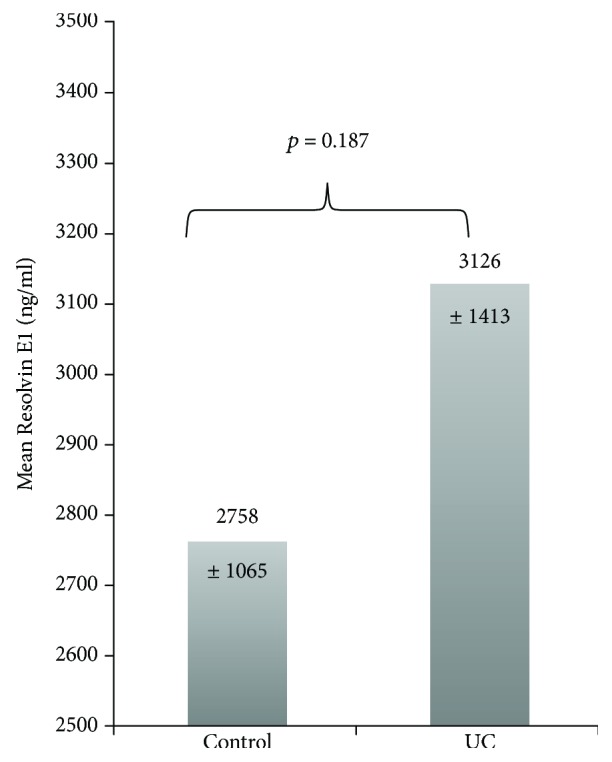
Comparison of serum Resolvin E1 levels of the patients with ulcerative colitis and control group.

Serum Resolvin E1 levels were measured in the UC group (3126 ± 1413 ng/ml) and in the control group (2758 ± 1065 ng/ml) (*p* = 0.187) (Figure 1). In the univariate analysis of the factors that may have an effect on the serum Resolvin E1 levels in the UC patient group, disease activity, UC localization, treatment methods, and gender did not show significant differences when compared to serum Resolvin E1 levels (Table 2).

**Figure 2:**
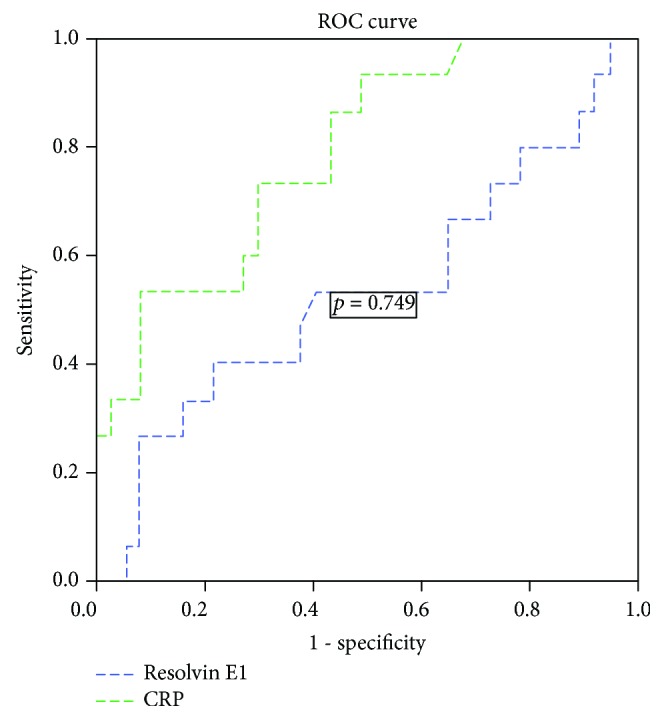
Efficiency of Resolvin E1 and CRP levels for the assessment of disease activity.

When the relationship between disease activity, serum Resolvin E1, and CRP levels were examined, serum Resolvin E1 levels did not differ significantly in the active and remitted UC patients. CRP levels were significantly higher in active UC patients (Table 3). In the correlation analysis between Resolvin E1, p-MS, and CRP value, a low-grade positive significant association was found between Resolvin E1 and CRP (*r* = 0.303, *p* = 0.031). There was no significant association between Resolvin E1 and p-MS (*r* = −0.207, *p* = 0.146). According to the ROC analysis of the inflammatory mediators that are evaluated in the study to determine the efficacy of UC disease activity, CRP was found to be effective, while Resolvin E1 was not (Figure 2).

## 4. Conclusion

Recent changes in the treatment and follow-up of IBD (inflammatory bowel disease) patients have been based on the better understanding of the mechanisms involved in the pathogenesis of the disease. The identification of cytokines, which are the basis of inflammation, has also affected treatment strategies.

As stated in the guidelines of ECCO (European Crohn's and Colitis Organization), the aim of treatment in IBD is not to control the symptoms of the disease but to prevent the underlying expression [11]. In clinical practice, this condition can be defined as mucosal healing [12, 13]. Endoscopic procedures provide adequate information for the evaluation of mucosal healing, but endoscopy is an invasive and expensive procedure. Therefore, it has encouraged researchers to develop easier and noninvasive methods to determine the severity of inflammation.

For this purpose, many biochemical and serological markers have been studied to determine the severity of inflammation in IBD [14, 15]. Today, the most commonly used inflammatory marker for detecting disease activity and the severity of inflammation in IBD is CRP and fecal calprotectin [16]. There are studies suggesting that CRP is highly compatible with the clinical activity of the disease, severity of endoscopic lesions, and the degree of inflammation in IBD [17, 18]. However, CRP gene polymorphism differs between individuals, and also, another factors such as increased serum levels in all acute phase conditions are factors that limit the use of CRP in IBD follow-up [2]. There are studies indicating that CRP is not always compatible with disease activity in IBD [19]. In the studies, Resolvin E1 produced from omega-3 EPA has strong anti-inflammatory effects both in *in vivo* and *in vitro* environments, and it has been found to play an important role in the resolution of inflammation [20–22]. In animal studies of colitis models, Resolvin E1 has been shown to reduce the severity of inflammation and to be effective in the regression of colitis [6, 23]. Resolvin E1 plays an important role in inhibiting the release of major cytokines in the pathogenesis of IBD such as TNF-alpha and IL12 in the development of anti-inflammatory activity [24, 25]. In addition, results show that Resolvin E1-induced intestinal alkaline phosphatase as an endogenous surface expressed factor is important in inflammatory resolution in the mucosa [26].

In this study, the serum Resolvin E1 levels were found to be higher in UC patients compared to the control group, but this difference was not statistically significant. Serum Resolvin E1 levels were not significantly different in active and remission term in UC patients. This may be explained by the fact that the number of patients included in the study was low and the severity of the disease in the active period was represented by mild or moderated activity. In assessing the efficacy of Resolvin E1 in predicting disease activity in UC, Resolvin E1 did not show a positive or strong correlation with CRP and p-MS. Therefore, Resolvin E1 is not considered to be a sufficient marker to demonstrate disease activity in UC. There are some limitations in our study. Total Mayo score was not used instead of p-MS in determining disease activity. In addition, the patients in the active period were not grouped according to the activity severity which is considered a missing aspect of the study. Furthermore, the strength of our study could be increased by measuring mucosal Resolvin E1 levels in addition to serum Resolvin E1 levels.

In another aspect, the possible effects of potent anti-inflammatory and immunosuppressive drugs used in the treatment of UC on Resolvin E1 levels should also be considered.

When the data from our study were gathered together with the information in the literature, Resolvin E1 may be an important marker of UC due to its endogenous lipid mediator effects and anti-inflammatory properties involved in the resolution of inflammation. However, the association of Resolvin E1 with disease activity in IBD is not as strong as the valuable markers of disease severity and inflammation, such as CRP or TNF-alpha.

## Figures and Tables

**Table 1:** Clinical and demographic characteristics of UC patients.

	Total (*n* = 51)		Active (*n* = 15)		Remission (*n* = 36)	
Disease duration years (median, min-max)	5	1-20	3	1-10	5	1-20
p-MS (median, min-max)	1	0-8	5	3-8	0	0-2
Localization (*n*, %)						
Proctitis	13	25.5	3	20.0	10	27.8
Left colitis	15	29.4	5	33.3	10	27.8
Extensive colitis	23	45.1	7	46.7	16	44.4
Treatment (*n*, %)						
ASA	30	58.8	8	53.3	22	61.1
AZA	1	2.0	0	0.0	1	2.8
Anti-TNF	5	9.8	1	6.7	4	11.1
Steroid	4	7.8	2	13.3	2	5.6
ASA+AZA	9	17.6	3	20.0	6	16.7
AZA+Anti-TNF	1	2.0	1	6.7	0	0.0
ASA+AZA+Anti-TNF	1	2.0	0	0.0	1	2.8

p-MS: partial Mayo score; ASA: aminosalicylic acid; AZA: azathioprine; Anti-TNF: anti-tumor necrosis factor.

**Table 2:** The effects of disease characteristics and treatment methods on serum Resolvin E1 level (ng/ml).

	*N*	Mean	SD	*p*
Disease activity				
Actıve	15	3132	1520	0.749
Remission	36	3114	1166	
Localization				
Proctitis	13	3069	1738	0.593
Left colitis	15	3172	1048	
Extensive colitis	23	3129	1479	
Treatment				
5-ASA	30	3338	1652	NA
AZA	1	1952	—	
Anti-TNF	5	2625	774	
AZA+Anti-TNF	1	4289	—	
Steroid	4	2973	1100	
ASA+AZA	9	2839	1008	
ASA+AZA+Anti-TNF	1	2504	—	

SD: standard deviation; ASA: aminosalicylic acid; AZA: azathioprine; TNF: tumor necrosis factor.

**Table 3:** Comparison of serum Resolvin E1 and CRP levels according to disease activity.

	Active		Remission		*p*
	Mean	SD	Mean	SD	
Resolvin E1 (ng/ml)	3132	1520	3114	1166	0.749
CRP (mg/dl)	2.7	3.7	0.5	0.5	0.002

## Data Availability

The data (demographic features, laboratory findings, colonoscopic findings, Resolvin E1 results, and treatment modalities) used to support the findings of this study are available from the corresponding author (Süleyman Günay) upon request.

## Abbreviations

ASA: Aminosalicylic acid
AZA: Azathioprine
CRP: C-reactive protein
COX2: Cyclooxygenase-2
chemR23: Chemerin receptor 23
DSS: Dextran sulfate sodium
ECCO: European Crohn's and Colitis Organization
IBD: Inflammatory bowel disease
UC: Ulcerative colitis
PUFA: Polyunsaturated fatty acids
TNF: Tumor necrosis factor
p-MS: Partial Mayo scoring
omega-3 EPA: Omega-3 eicosapentaenoic acid
TNBS: Trinitrobenzenesulfonic acid
IL-12: Interleukin 12.
